# Treatment of heterotopic ossification via inhibiting the MMP-2/CDH5 axis through oral delivery of network pharmacology-predicted Chinese medicine

**DOI:** 10.1016/j.mtbio.2026.103107

**Published:** 2026-04-09

**Authors:** Junchao Huang, Jinxi An, Le He, Huajun Wang, Jiachang Hong, Ziheng Bu, Xudong Zhang, Wei Liu, Tingyu Wu, Seng Wang, Wei Zhu, Yuhui Li, Jixian Wan, Min Sun, Jianzhong Du, Peng Wu

**Affiliations:** a Department of Sports Medicine, Shanghai East Hospital, School of Medicine, Tongji University, Shanghai, 200120, PR China; b School of Materials Science and Engineering, East China University of Science and Technology, Shanghai, 200237, PR China; c Department of Sports Medicine, The First Affiliated Hospital, The Guangzhou Key Laboratory of Precision Orthopedics and Regenerative Medicine, Guangdong Provincial Key Laboratory of Speed Capability, Jinan University, Guangzhou, Guangdong, 510630, PR China; d Department of Orthopedics, Shanghai Tenth People's Hospital, School of Medicine, Tongji University, Shanghai, 200072, PR China

**Keywords:** Oral hydrogel, Heterotopic ossification, Matrix Metalloproteinase-2, Forsythoside A, Network pharmacology

## Abstract

Heterotopic ossification (HO) is characterized by ectopic bone formation in soft tissues such as tendons, with an incidence exceeding 30% in high-risk populations. Currently, no targeted therapeutic options are available. In this study, matrix metalloproteinase-2 (MMP-2) was identified as a critical promoter of HO by mediating the degradation of vascular endothelial Cadherin 5 (CDH5), thereby enhancing vascular permeability and facilitating inflammatory cell infiltration. Through network pharmacology analysis, Forsythoside A (FA), a compound derived from the traditional Chinese medicine *Forsythia*, was predicted to act as a potential MMP-2 inhibitor. However, the clinical application of FA is hindered by its narrow therapeutic window. To overcome this limitation, we developed an intestinal enzyme-responsive hydrogel (FA@SD-hydrogel) for targeted delivery of FA. This delivery system significantly improved the bioavailability of FA and enabled sustained release, thereby reducing the required dosage and associated toxicity. Comprehensive *in vivo* and *in vitro* experiments demonstrated that FA@SD-hydrogel achieved superior therapeutic efficacy against HO with reduced toxicity. By inhibiting MMP-2, the hydrogel effectively suppressed ectopic bone formation, reduced local vascular permeability, and reversed the inflammatory microenvironment driven by M1 macrophages, ultimately reprogramming the osteogenic niche. Together, these findings underscore the critical role of the MMP-2/CDH5 axis in HO pathogenesis and establish a targeted therapeutic strategy *via* FA@SD-hydrogel through the integration of network pharmacology and biomaterial engineering.

## Graphical abstract

**Figure:**
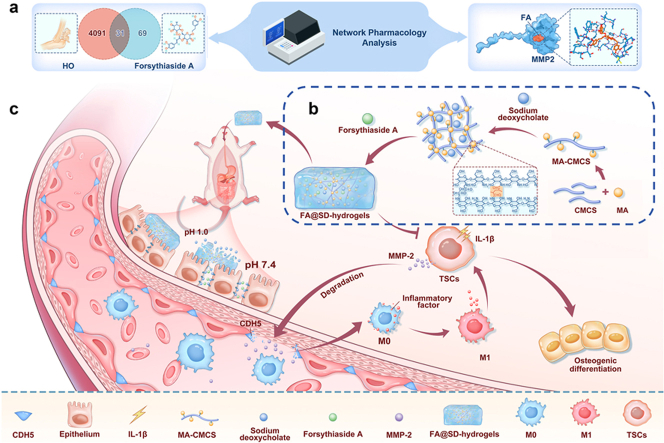

## 1 Introduction

Heterotopic ossification (HO), the pathological formation of bone in soft tissues, represents a significant clinical complication following traumatic injuries, joint surgeries, or neurologic insults. With a prevalence exceeding 30% in high-risk scenarios such as acetabular fracture fixation or total hip arthroplasty, HO severely impairs joint function and patient recovery [[1], [2], [3]]. The pathogenesis is driven by the inflammatory microenvironment-induced osteogenic differentiation of progenitor cells like tendon stem cells (TSCs) [4,5]. While current first-line prophylaxis with non-steroidal anti-inflammatory drugs (NSAIDs) aims to suppress this inflammation, its efficacy remains limited to approximately 60%, underscoring the involvement of alternative, NSAID-insensitive pathways. This highlights an urgent need for targeted therapeutics that can reprogram the osteogenic microenvironment rather than merely suppress inflammation [[6], [7], [8]].

Among the multifaceted mechanisms of HO, the matrix metalloproteinase family plays a pivotal role in inflammatory regulation and tissue remodeling [9]. This study focuses on matrix metalloproteinase-2 (MMP-2), which we identified as a key mediator in HO progression. We propose a novel mechanism wherein MMP-2, upregulated in the inflammatory milieu, degrades Cadherin 5 (CDH5), a critical adhesive junction protein essential for vascular endothelial integrity [[10], [11], [12], [13]]. This degradation compromises vascular barrier function, facilitating the chemotaxis and extravasation of inflammatory cells into soft tissues, thereby exacerbating the local inflammation that fuels heterotopic bone formation. Network pharmacology employs systems-based computational approaches to systematically predict compound-target interactions by constructing drug-target-disease networks, enabling rapid screening of potential therapeutics and mechanistic elucidation. This methodology integrates molecular databases and bioinformatics analysis to efficiently identify key pathological targets and their regulatory pathways, providing a theoretical framework for targeted drug discovery. Through network pharmacology analysis, we subsequently identified Forsythoside A (FA), a natural compound with documented anti-inflammatory properties [14,15], as a potential inhibitor targeting this MMP-2/CDH5 axis.

To translate this finding into a viable therapy, we addressed the challenge of FA's side effects associated with systemic administration by designing an advanced intestinal enzyme-responsive hydrogel delivery system. This material innovation involves the synthesis of a methacrylated carboxymethyl chitosan-based hydrogel co-loaded with FA and sodium deoxycholate, forming FA@SD-hydrogel upon UV cross-linking. This smart hydrogel is engineered to enhance intestinal absorption and achieve the sustained, synchronized release of both agents specifically in the intestine, thereby maximizing local drug efficacy while significantly reducing the required systemic dose and minimizing adverse effects.

**Scheme 1:**
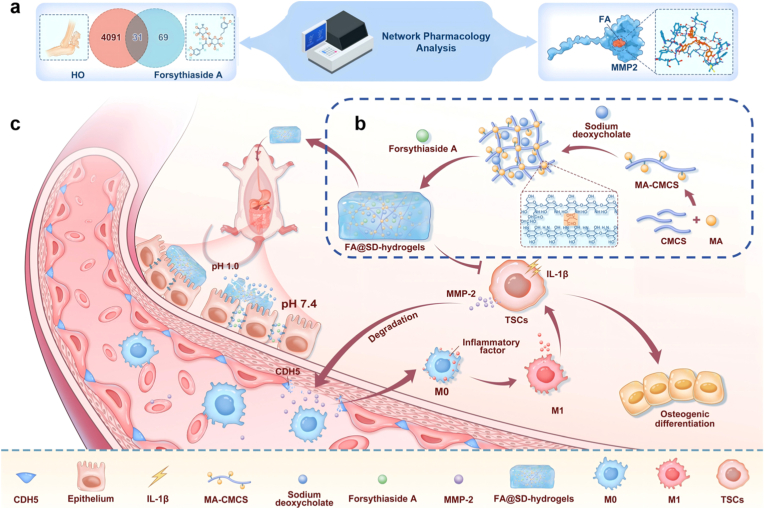
a) Schematic illustration of network pharmacology analysis and molecular docking between Forsythoside A and MMP-2. The Venn diagram intersects HO-related genes and FA target genes, identifying 31 common targets. Subsequent analysis highlighted the MMP family (particularly MMP-2) as key hub genes. Molecular docking revealed strong binding affinity between FA and the MMP-2 protein. b) Synthetic scheme of FA@SD-hydrogel. The hydrogel is fabricated by photo-initiated crosslinking of methacrylated carboxymethyl chitosan (MA-CMCS) co-loaded with FAand sodium deoxycholate (SD) under UV irradiation, forming an intestinal enzyme-responsive drug delivery system. c) Mechanistic diagram illustrating the therapeutic action of FA@SD-hydrogel against HO *in vitro* and *in vivo*. In the inflammatory microenvironment, IL-1β-stimulated TSCs secrete MMP-2, which degrades endothelial CDH5, increasing vascular permeability and promoting M1 macrophage infiltration. FA@SD-hydrogel responds to intestinal pH and lysozyme, releasing FA to inhibit MMP-2 expression, restore CDH5 integrity, shift macrophage polarization from M1 to M2 phenotype, and ultimately repress osteogenic differentiation.

The primary objective of this study is to evaluate the therapeutic potential of FA for HO, specifically through its regulation of the MMP-2/CDH5 axis. Network pharmacology predicted high-affinity FA-MMP-2 binding (−8.867 kcal/mol), yet experimental validation revealed a narrow therapeutic window: while 400 μg/mL FA effectively suppressed MMP-2, it induced significant cytotoxicity *in vitro* and caused multi-organ damage in rats upon oral administration (20 mg/kg for 14 days). To overcome this limitation, we engineered an intestinal enzyme-responsive FA@SD-hydrogel that halves the total drug dosage while achieving sustained 48 h intestinal release. This biomaterial-based platform maintains therapeutic FA levels, avoids bolus-induced toxicity, and demonstrates superior efficacy to oral FA—significantly reducing ectopic bone volume, restoring endothelial CDH5 integrity, and reversing M1/M2 macrophage imbalance in HO models. By integrating the discovery of a novel MMP-2/CDH5 pathogenic axis with a clinically translatable drug delivery strategy, our work establishes FA@SD-hydrogel as a safer, more effective paradigm for HO prevention and treatment (Scheme 1).

## 2 Results

### 2.1 FA may exert therapeutic effects against HO by targeting MMP-2

**Fig. 1:**
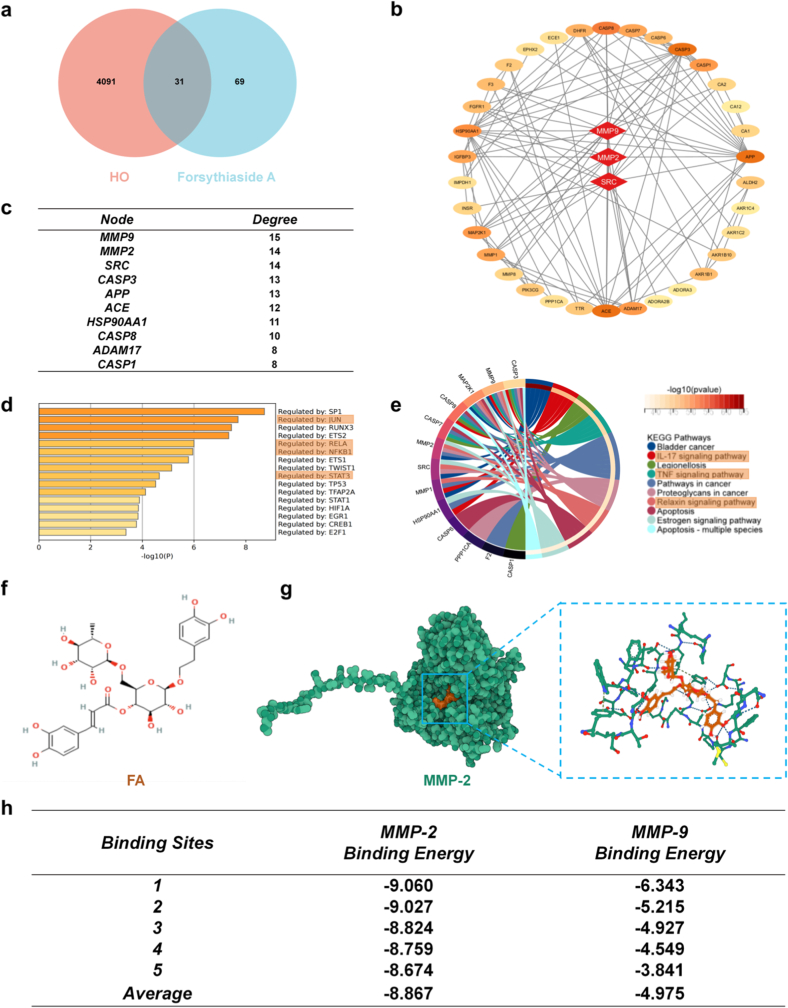
Network pharmacological analysis reveals that FA may treat HO by targeting MMP-2. a) Venn diagram of FA target genes and HO-related genes, showing 31 common targets. b) Topological analysis of the PPI network indicates significant enrichment of the MMPs family. c) Degree value ranking of the key targets. d) Enrichment of transcription factors corresponding to the common targets. e) KEGG pathway enrichment analysis of the common targets. f) Molecular structure of FA. g) Molecular docking model depicting the binding mode of FA to the MMP-2 protein. h) Binding energy distribution diagram of FA with MMP-2 (average binding energy = −8.867 kcal/mol) or MMP-9 (average binding energy = −4.975 kcal/mol).

We planned to use network pharmacology analyses to investigate whether FA has potential therapeutic effects on HO. By performing a Venn analysis of 4122 HO-related genes from the CTD database and 100 FA target genes from the SwissTargetPrediction database (Fig. 1a), we identified 31 intersecting genes. A PPI network was constructed for these 31 intersecting genes (Fig. S1). Topological analysis of the PPI revealed significant enrichment of the MMP family (Fig. 1b), with MMP-9 and MMP-2 ranking first and second in centrality, respectively (Fig. 1c). We profiled the transcription factors associated with the intersecting genes and found enrichment of JUN, STAT3, RELA, and NFKB1—factors implicated in inflammation, osteogenesis, and cell proliferation (Fig. 1d). We then performed GO and KEGG analyses on the intersecting genes. KEGG analysis showed significant enrichment of the TNF, IL-17, and relaxin signaling pathways. The canonical inflammatory TNF pathway can directly activate NF-κB signaling and mediate apoptosis [16]; the IL-17 pathway regulates the expression of proinflammatory mediators and antimicrobial peptides and can indirectly activate NF-κB [17]; the relaxin pathway modulates extracellular matrix remodeling, collagen metabolism, and osteogenic differentiation, and activates the TGF-β/BMP–Smad pathway to promote osteoblast differentiation and drive soft tissue calcification [18] (Fig. 1e). GO-CC indicated that the gene products are significantly associated with the extracellular matrix. These findings suggest that FA primarily treats HO by targeting inflammation-related factors and extracellular matrix–related components (Fig. S2). Among the 31 common genes, the MMP family—key regulators of the extracellular matrix—drew particular attention. MMP-9 is a classic marker of HO, whereas the mechanisms of MMP-2 in HO are less studied and merit focused investigation. MCODE can detect tightly connected gene clusters within complex biological networks and reveal gene–gene interactions. Our MCODE results showed that MMP-2 is tightly connected with most hub genes (Fig. S3), suggesting a central role for MMP-2 in FA-mediated regulation. Accordingly, we retrieved the chemical structure of FA (Fig. 1f) and conducted molecular docking (Fig. 1g) including FA-MMP-2 and FA-MMP-9. In molecular docking, a binding energy lower than −4.0 kcal/mol indicates a potential interaction, while lower than −6.0 kcal/mol suggests good binding affinity. The average binding energy between FA and MMP-2 was −8.867 kcal/mol, indicating excellent binding potential. In contrast, the average binding energy between FA and MMP-9 was only −4.975 kcal/mol, suggesting a weak interaction with significantly lower binding potential (Fig. 1h).

### 2.2 The effective concentration of FA for regulating MMP-2 is close to its toxic concentration

**Fig. 2:**
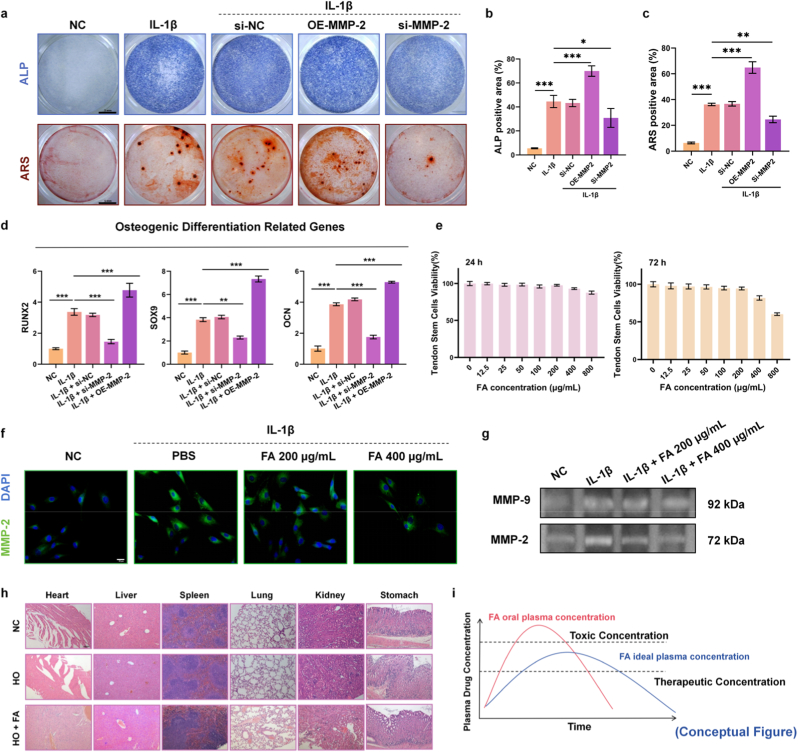
FA exhibits proximal effective and toxic concentrations while modulating MMP-2. a) Alkaline phosphatase (ALP) and Alizarin Red S (ARS) staining of TSCs under different treatments. Scale bar:5 mm. b) Quantitative analysis of ALP activity and c) mineralized nodule formation. Data are expressed as means ± SD (N = 3, ∗*p* < 0.05, ∗∗*p* < 0.01, ∗∗∗*p* < 0.001). d) mRNA expression levels of chondrogenic (SOX9) and osteogenic (RUNX2, OCN) markers in TSCs under different treatment conditions. Data are expressed as means ± SD (N = 3). e) Viability of TSCs after 24 h or 72 h of incubation with different concentrations of FA. Data are expressed as means ± SD (N = 3). f) Immunofluorescence staining of MMP-2 protein in TSCs under different treatment conditions. Scale bar: 20 μm. g) Representative gelatin zymography of MMP-9 and MMP-2 activities in TSCs under different treatment conditions. h) H&E-stained sections of major organs (heart, liver, spleen, lung, kidney, stomach) from rats in different treatment groups. Scale bar: 100 μm. i) Conceptual schematic of the simulated relationship between FA blood concentration over time and its toxic and therapeutic thresholds. (For interpretation of the references to colour in this figure legend, the reader is referred to the Web version of this article.)

To validate the network pharmacology findings, we first isolated and identified TSCs) using our previously established methods (Fig. S4) [19]. Subsequently, we generated MMP-2 overexpression and knockdown plasmids and validated their efficiencies (Fig. S5 and S6). Alkaline phosphatase (ALP) and Alizarin Red S (ARS) staining showed (Fig. 2a) that IL-1β induced an osteogenic differentiation tendency in these TSCs. MMP-2 overexpression markedly enhanced induced osteogenesis, whereas MMP-2 knockdown reversed this trend (Fig. 2b and c). Following MMP-2 overexpression, transcript levels of the chondrogenic marker SOX9 and osteogenic markers RUNX2 and OCN were significantly upregulated in TSCs, while MMP-2 knockdown yielded the opposite effect (Fig. 2d). These results indicate that MMP-2 plays a pivotal role in the progression of HO, and its inhibition can mitigate the osteogenic tendency. To determine a safe dosing range for FA, we assessed FA cytotoxicity in rat TSCs at 24 h, 48 h (Fig. S7), and 72 h using the Cell Counting Kit-8 (CCK-8) assay. At 72 h, cell viability was not markedly reduced at 200 μg/mL, whereas co-incubation at 400 μg/mL produced pronounced cytotoxicity (Fig. 2e). We performed immunofluorescence at 200 and 400 μg/mL FA to evaluate its targeting of MMP-2 (Fig. 2f). FA at 400 μg/mL suppressed MMP-2 expression more strongly than at 200 μg/mL (Fig. S8), indicating greater toxicity alongside stronger MMP-2 modulation at 400 μg/mL. Furthermore, to evaluate the direct functional impact of FA, we conducted a gelatin zymography assay. Consistent with our molecular docking predictions, the results demonstrated that FA significantly suppressed the enzymatic activity of MMP-2 in a dose-dependent manner, whereas the activity of MMP-9 was only marginally affected compared to the robust inhibition observed for MMP-2. (Fig. 2g, Fig. S9). Compared to the normal control (NC) and HO model groups, H&E staining revealed that oral administration of FA (20 mg/kg daily for 14 days), whether administered alone (FA group) or after HO induction (HO + FA group), caused varying degrees of damage to key organs, including the heart, liver, spleen, lung, kidney and stomach (Fig. 2h). Collectively, these data suggest that, *via* oral administration, the effective concentration of FA is close to its toxic threshold. To preserve efficacy without eliciting toxicity, the delivery route should be optimized to maintain therapeutic plasma levels over time while remaining below the safety limit (Conceptual Figure, Fig. 2i).

### 2.3 FA@SD-hydrogel enhances intestinal absorption of FA and exhibits good biocompatibility and intestinal retention

**Fig. 3:**
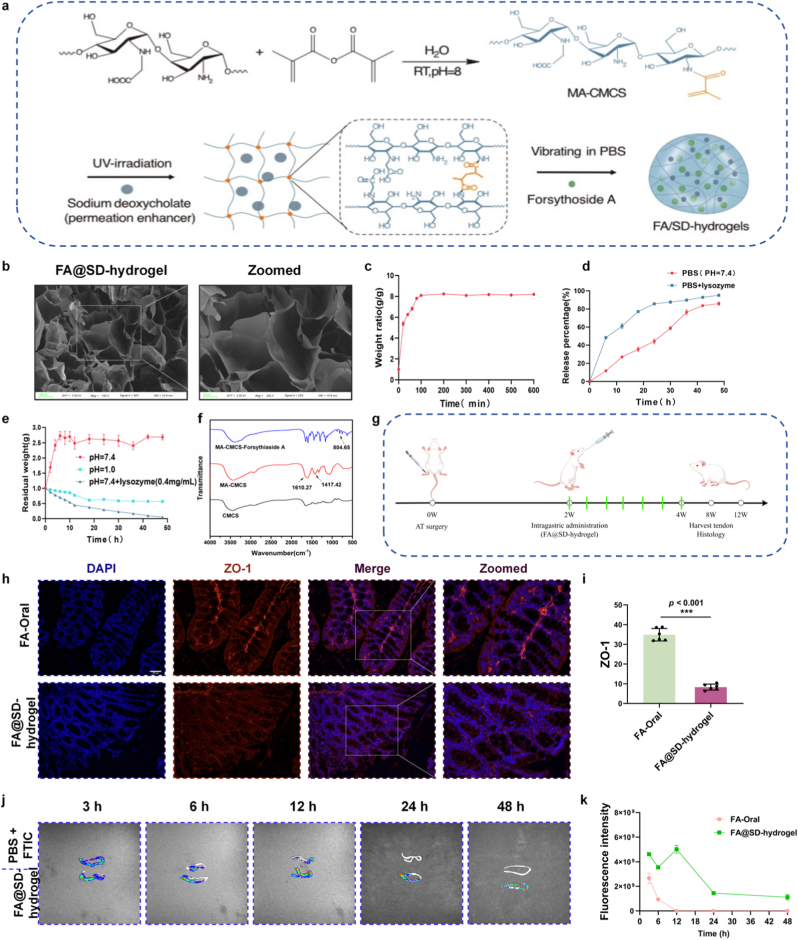
Construction, characterization, intestinal retention and absorption enhancement function of FA@SD-hydrogel. a) Schematic diagram of FA@SD-hydrogel construction and its FA sustained release mechanism in response to intestinal environment (alkaline pH and lysozyme). b) Scanning electron microscopy (SEM) image of FA@SD-hydrogel. c) Swelling performance of FA@SD-hydrogel in different pH environments. Data are expressed as means ± SD (N = 3). d) *in vitro* cumulative release profile of FA from FA@SD-hydrogel in PBS buffer (pH 7.4) with or without lysozyme. Data are expressed as means ± SD (N = 3). e) Degradation behavior of FA@SD-hydrogel under different treatment conditions. Data are expressed as means ± SD (N = 3). f) Fourier transform infrared (FT-IR) spectra of MA-CMCS, FA, SD and FA@SD-hydrogel. g) Schematic diagram of the administration protocol for FA@SD-hydrogel in animal experiments. h) Representative immunofluorescence staining images of ZO-1 protein in intestinal tissues from different treatment groups. Nuclei were stained with DAPI. Scale bar: 50 μm. i) Quantitative analysis of ZO-1 fluorescence intensity. Data are expressed as means ± SD (N = 5). j) Representative *in vivo* fluorescence images of FA@SD-hydrogel retention in rat intestine at different time points after oral administration and k) corresponding quantitative analysis of fluorescence intensity. Data are expressed as means ± SD (N = 3, ∗*p* < 0.05, ∗∗*p* < 0.01, ∗∗∗*p* < 0.001).

To address the above issues with FA, we aimed to increase its absorption efficiency and achieve sustained and controlled release, thereby reducing the total dose, keeping plasma FA levels within a controllable range, and preventing toxic concentrations. Accordingly, we constructed the FA@SD-hydrogel based on an intestine-targeted drug delivery hydrogel system previously established by our research group [20]. SD can disrupt the tight junction protein ZO-1 between intestinal epithelial cells, improving drug delivery and enhancing FA absorption. The hydrogel is formed by photo-initiated crosslinking of glycidyl methacrylate–modified carboxymethyl chitosan (MA-CMCS). MA-CMCS adheres well to the intestinal mucosa, improving the intestinal residence of FA@SD-hydrogel. In addition, glycosidic bonds on MA-CMCS chains are hydrolyzed by intestinal lysozyme, and CMCS itself degrades slowly in mildly alkaline environments, enabling FA@SD-hydrogel to respond specifically to intestinal conditions and degrade to release FA in a sustained manner (Fig. 3a). Electron microscopy revealed a porous architecture with heterogeneous pore sizes, rough surfaces, and relatively uniform distribution. This porosity supports drug loading and release and promotes interactions with the intestinal mucosa (Fig. 3b, Fig. S10). Owing to the hydrophilicity of MA-CMCS, FA@SD-hydrogel exhibited favorable swelling characteristics (Fig. 3c). Sustained-release assays showed robust FA release from FA@SD-hydrogel in response to intestinal pH value and enzymes, with complete release within 48 h (Fig. 3d). Crucially, FA@SD-hydrogel did not exhibit sustained-release function in the low-pH gastric environment; this property was only activated upon entry into the small intestine, where neutral pH and intestinal enzymatic activity cooperated to induce hydrogel degradation and sustained FA release. (Fig. 3e). FTIR analysis confirmed successful encapsulation of FA and SD within FA@SD-hydrogel (Fig. 3f). The dosing regimen for FA@SD-hydrogel starting in week 2 post-modeling, administer once every two days for two weeks (Fig. 3g). In FA@SD-hydrogel, MA-CMCS's mucosal adhesion and its suppression of the tight junction protein ZO-1 (Zonula Occludens-1) help retain the hydrogel in the intestine and enhance FA absorption, maintaining effective drug levels. ZO-1 is essential for barrier integrity; its inhibition increases macromolecular permeability [21]. Immunofluorescence demonstrated that FA@SD-hydrogel suppresses intestinal ZO-1, opening paracellular gaps and improving FA absorption efficiency (Fig. 3h and i). In rat intestinal retention studies, a substantial amount of hydrogel remained in the gut at 48 h (Fig. 3j and k), likely attributable to the strong mucosal adhesion of MA-CMCS. Together, these results indicated that FA@SD-hydrogel provides efficient FA delivery and robust intestinal residence.

### 2.4 FA@SD-hydrogel exhibits lower toxicity and superior therapeutic efficacy

**Fig. 4:**
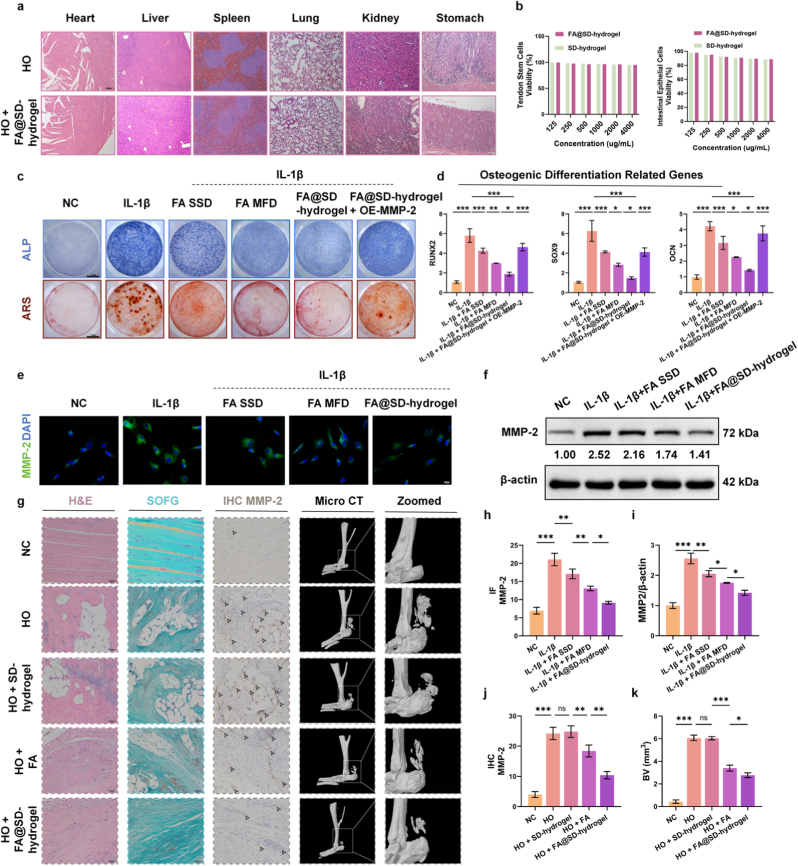
*In vitro* and *in vivo* evaluation of the biocompatibility and therapeutic efficacy of FA@SD-hydrogel. a) Representative H&E-stained images of major organs (heart, liver, spleen, lung, kidney, stomach) from rats after different treatments. Scale bar: 100 μm. b) Viability of TSCs (left) and IEC-6 (right) after incubation with SD-hydrogel or FA@SD-hydrogel. Data are expressed as means ± SD (N = 3). c) Representative images of ALP and ARS staining of TSCs in different treatment groups. Scale bar: 5 mm. d) Relative mRNA expression levels of chondrogenic (SOX9) and osteogenic (RUNX2, OCN) markers in TSCs after different treatments. Data are expressed as means ± SD (N = 3). e) Representative immunofluorescence images of MMP-2 protein in TSCs after different treatments. Nuclei were stained with DAPI. Scale bar: 20 μm. f) Representative Western Blot bands and relative quantification of MMP-2 protein expression in TSCs after different treatments. g) Representative images of H&E staining, Safranin O/Fast Green (SOFG) staining, and MMP-2 immunohistochemical (IHC) staining of tendon tissues from the HO rat model (left panel) Scale bar: 50 μm. Three-dimensional reconstructed micro-computed tomography (Micro-CT) images of the Achilles tendon region in rat hindlimbs (right panel). h) Quantitative analysis of MMP-2 immunofluorescence intensity and i) protein expression in TSCs after different treatments. Data are expressed as means ± SD (N = 3). j) Quantitative analysis of MMP-2 immunohistochemistry and k) heterotopic bone volume in the hindlimbs of HO rats. Data are expressed as means ± SD (N = 3). (For interpretation of the references to colour in this figure legend, the reader is referred to the Web version of this article.)

After 10 weeks of feeding administration in the HO rat model, to comprehensively evaluate systemic safety, we included Sham groups, SD-hydrogel-only groups, FA@SD-hydrogel-only groups, HO groups and HO + FA@SD-hydrogel groups. H&E staining of major organs (heart, liver, spleen, lung, kidney and stomach) showed no significant pathological damage across these control groups, confirming the excellent biocompatibility of the hydrogel carrier itself (Fig. 4a and Fig. S11). This is attributable to the fact that over the 2-week treatment window, the total FA dose delivered by FA@SD-hydrogel was only half that of oral FA, and its sustained-release property effectively controlled FA release, avoiding the adverse effects associated with bolus high-dose exposure. CCK-8 proliferation assays indicated that neither SD-hydrogel nor FA@SD-hydrogel exerted significant antiproliferative effects on TSCs and rat intestinal epithelial cells (IEC-6) at concentrations up to 4000 μg/mL (Fig. 4b). The concentration was defined based on the mass of the SD-hydrogel excluding the loaded FA. Next, we compared its therapeutic efficacy against HO with that of direct FA administration. *in vitro* ARS and ALP assays showed that both a single standard dose of FA (FA SSD) and FA@SD-hydrogel suppressed IL-1β–induced osteogenic differentiation in TSCs, with FA@SD-hydrogel exerting a stronger inhibitory effect. Moreover, a multiple fractional doses group (FA MFD) exhibited an inhibitory effect intermediate between FA SSD and FA@SD-hydrogel, with FA@SD-hydrogel exerting the strongest inhibitory effect. Notably, overexpressing MMP-2 during FA@SD-hydrogel treatment abrogated its therapeutic effect (Fig. 4c, Fig. S12). RT-qPCR results demonstrated that FA@SD-hydrogel significantly decreased transcriptional activation of the chondrogenic marker SOX9 and osteogenic markers RUNX2 and OCN; however, concurrent MMP-2 overexpression reversed the inhibition of osteogenic differentiation by FA@SD-hydrogel (Fig. 4d). Immunofluorescence indicated that FA@SD-hydrogel effectively suppressed MMP-2 expression (Fig. 4e and h), a trend corroborated by Western blotting (Fig. 4f and i). H&E staining of HO model sections showed that from 12 weeks post-modeling, rat tendons progressively developed chondrogenic and osteogenic foci, accompanied by neovascular ingrowth, disordered tendon architecture, and inflammation, both oral FA and FA@SD-hydrogel markedly reversed these phenotypes, with FA@SD-hydrogel yielding superior outcomes. Safranin O/Fast Green (SOFG) staining revealed increased proteoglycan content, matrix chondrogenesis, and neovascularization in rat tendons after HO induction, particularly at week 12. FA@SD-hydrogel attenuated osteogenic changes and outperformed oral FA (Fig. 4g), IHC in the HO model showed that both oral FA and FA@SD-hydrogel effectively suppressed MMP-2 expression, with a stronger inhibitory effect observed for FA@SD-hydrogel (Fig. 4g and j). Micro-CT of rat hindlimbs demonstrated that FA@SD-hydrogel significantly reduced the volume of ectopic bone at the Achilles tendon, outperforming oral FA (Fig. 4k). Notably, the HO + SD-hydrogel group exhibited no significant therapeutic effects across these evaluations, confirming that the observed efficacy is specifically attributable to FA rather than the hydrogel matrix. Compared with FA alone, FA@SD-hydrogel maintains a higher local concentration of FA, thereby achieving more effective treatment of HO.

### 2.5 Transcriptomic profiling and clinical validation of the MMP-2/CDH5 axis in HO

**Fig. 5:**
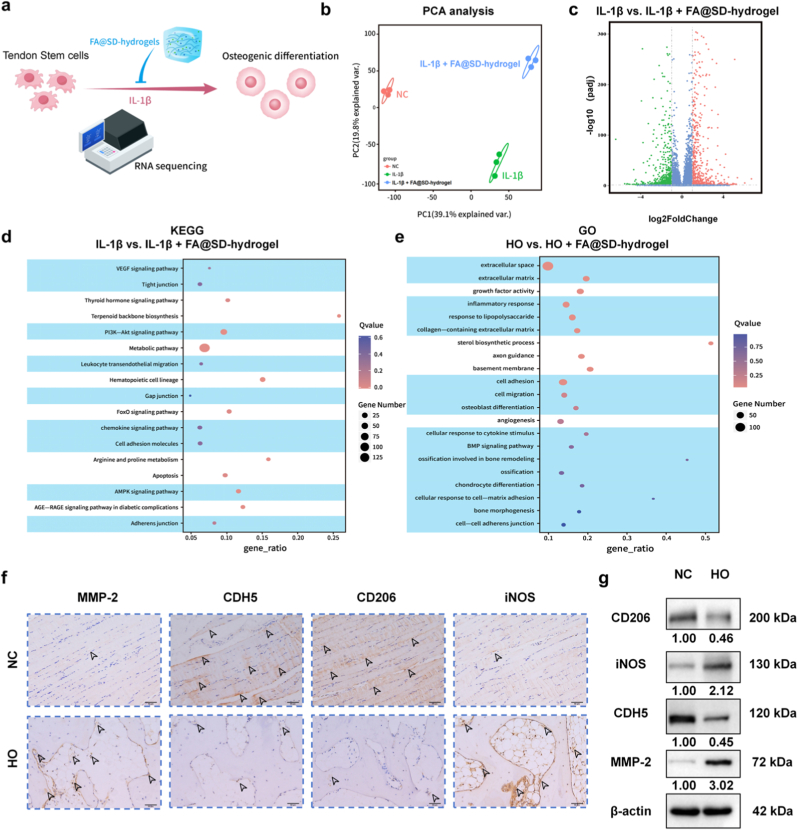
*In vitro* transcriptomic alterations and clinical pathological features of HO. a) Schematic diagram of the mRNA sequencing workflow for TSCs under different treatments. b) Principal component analysis (PCA) of the sequencing data. c) Volcano plot of differentially expressed genes between IL-1β and IL-1β + FA@SD-hydrogel. d) KEGG pathway enrichment analysis of differentially expressed genes. e) GO functional enrichment analysis of differentially expressed genes. f) Representative immunohistochemical (IHC) staining of MMP-2, CDH5, CD206 and iNOS in human tissues from healthy individuals and HO patients. Scale bar: 50 μm. g) Western blot analysis of MMP-2, CDH5, CD206, and iNOS protein levels in human tissues from healthy individuals and HO patients.

To further elucidate the molecular mechanisms underlying FA@SD-hydrogel's therapeutic effects, we performed RNA sequencing on *in vitro* cultured rat TSCs from control (NC groups), IL-1β-stimulated model (IL-1β groups), and IL-1β-stimulated cells treated with FA@SD-hydrogel groups (IL-1β + FA@SD-hydrogel groups) (Fig. 5a). Principal component analysis (PCA) showed clear separation among the three groups, indicating distinct transcriptional profiles (Fig. 5b). Volcano plot analysis comparing IL-1β vs. IL-1β + FA@SD-hydrogels showed significant differential expression of inflammation- and osteogenesis-related genes (Fig. 5c). KEGG and GO enrichment analyses revealed that following administration of FA@SD-hydrogel, multiple pathways were enriched, including those associated with M1/M2 polarization (e.g., inflammatory response, response to lipopolysaccharide, cellular response to cytokine stimulus, Chemokine signaling pathway), osteogenesis (e.g., osteoblast differentiation, ossification involved in bone remodeling, ossification, BMP signaling pathway), and adhesion (e.g., Adherens junction, cell adhesion, cell migration, Leukocyte transendothelial migration). These enrichment results guided the direction of our mechanistic investigation of FA@SD-hydrogel. (Fig. 5d and e).

CDH5 is a type II classical cadherin primarily expressed in vascular endothelial cells and plays a central role in maintaining vascular structure and function. In oncologic research, the MMP family and the endothelial cadherin CDH5 act in critical concert: MMPs degrade the extracellular matrix to create space for tumor invasion and recruit macrophages, promoting their local migration and polarization [22]. MMPs can also downregulate CDH5 expression, increasing local vascular permeability and thereby chemotactically driving leukocyte trafficking—particularly macrophages—into the tumor inflammatory microenvironment [23,24], accelerating tumor progression. Our studies indicate that HO likewise involves excessive MMP secretion and remodeling of the local inflammatory microenvironment; therefore, we sought to determine whether the MMP–CDH5 synergy operates in HO as well, ultimately facilitating the recruitment of inflammatory mediators to the lesion and exacerbating HO progression. To this end, we collected elbow joint tissues from healthy individuals and patients with HO. Immunohistochemistry revealed high MMP-2 expression and low CDH5 levels in HO clinical specimens. Concurrently, the expression of the M2 macrophage polarization marker CD206 was notably low, whereas inducible nitric oxide synthase (iNOS) was highly expressed (Fig. 5f). Furthermore, the differential expression of CD206, iNOS, CDH5 and MMP-2 was corroborated *via* Western blot analysis of the clinical specimens. (Fig. 5g, Fig. S13). Collectively, these findings substantiate a pathogenic role for MMP-2 in HO and suggest that MMP–CDH5 cooperation likely operates in this context: MMP-2-mediated degradation of CDH5 may increase local vascular permeability, promote the influx of inflammatory mediators into the lesion, drive macrophage polarization toward the M1 phenotype, and aggravate the inflammatory microenvironment of HO.

### 2.6 FA@SD-hydrogel stabilizes vascular permeability by targeting the degradation of MMP-2, thereby reversing the formation of the inflammatory microenvironment

**Fig. 6:**
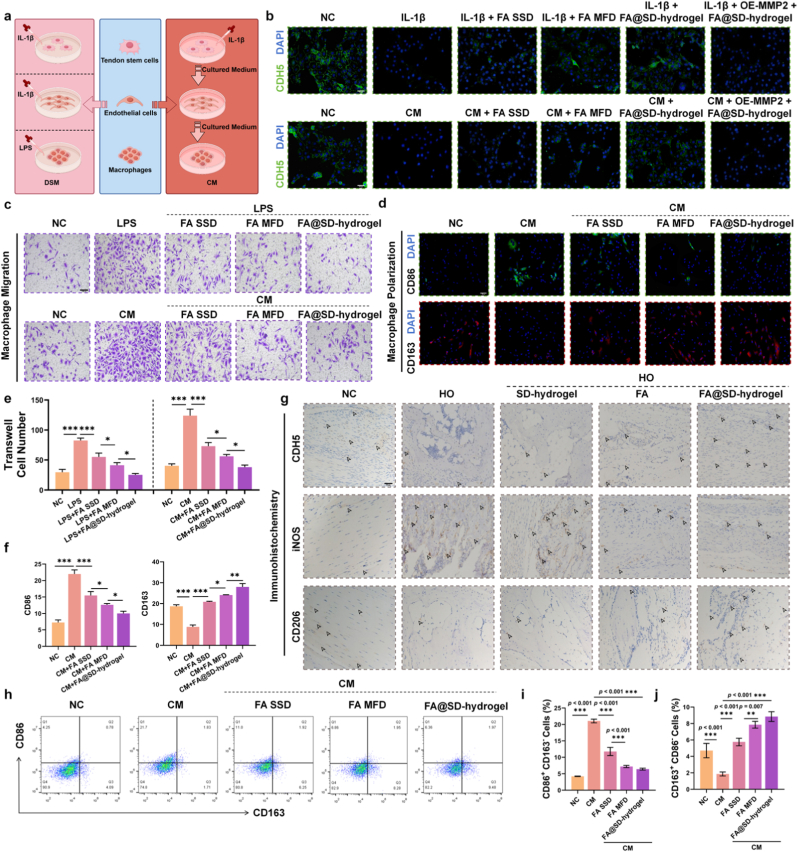
FA@SD-hydrogel improves the inflammatory microenvironment by preserving CDH5 to maintain vascular integrity and by inhibiting macrophage M1 polarization. a) Schematic of the experimental design illustrating the roles of DSM and CM in modeling the full course of HO. b) Representative immunofluorescence images of the endothelial junction protein CDH5 after treatment with IL-1β or conditioned medium (CM) and intervention with FA or FA@SD-hydrogel. Scale bar: 50 μm. c) Representative light-microscopy images of macrophage migratory capacity after the indicated treatments. Scale bar: 100 μm. d) Confocal laser scanning microscopy images of CD163 and CD86 staining in macrophages following the indicated treatments. Scale bar: 50 μm. e) Quantitative analysis of macrophage migration assessed by Transwell assay. Data are expressed as means ± SD (N = 3). f) Quantitative analysis of CD163 and CD86 fluorescence intensity in macrophages under the indicated treatments. Data are expressed as means ± SD (N = 3). g) Representative IHC images of CDH5, iNOS, and CD206 in tendon tissues from rats with the HO model. Scale bar: 50 μm. h) Representative flow cytometry dot plots of macrophages stained with anti-CD86 and anti-CD206 antibodies across treatment groups. i) Quantitative analysis of CD86 and j) CD163 by flow cytometry.

According to the above sequencing results, alterations in local vascular adhesion junction leading to changes in macrophage activity within the microenvironment, play a pivotal role in the course of HO. To this end, we compared directly stimulated media (DSM; culture medium supplemented with IL-1β or LPS) and conditioned media (CM; specifically, cell-free supernatants were collected from IL-1β-stimulated TSCs to culture endothelial cells, and the resulting supernatants from these endothelial cells were subsequently harvested to stimulate bone marrow-derived macrophages [BMDMs]) regarding their differential regulation of CDH5 expression and macrophage activity, to delineate the full course of HO development (Fig. 6a).The results showed that both IL-1β and CM reduced the expression of the endothelial junctional protein CDH5 when applied to endothelial cells; treatment with FA SSD, FA MFD, and FA@SD-hydrogel reversed this effect, with FA@SD-hydrogel performing better. Notably, the protective effect of FA@SD-hydrogel on CDH5 was significantly abrogated by MMP-2 overexpression (IL-1β + OE-MMP2 + FA@SD-hydrogel), functionally confirming that CDH5 preservation is mediated *via* targeted MMP-2 inhibition (Fig. 6b, Fig. S14). We next compared the migratory and differentiation capacities of macrophages stimulated with LPS versus those cultured in endothelial cell supernatant–based CM. Compared with the LPS group, macrophages in CM displayed enhanced migration and a stronger propensity to polarize toward the M1 phenotype. This can be attributed to increased vascular permeability following CDH5 degradation, which facilitates trafficking of inflammatory cells to lesion sites and exacerbates the local inflammatory microenvironment. Similarly, FA SSD, FA MFD, and FA@SD-hydrogel reduced macrophage migratory capacity across culture conditions with FA@SD-hydrogel performing better (Fig. 6c and e). In the *in vitro* polarization assays, while FA SSD showed limited efficacy, simulated sustained release *via* FA MFD significantly improved the modulation of macrophage markers, though FA@SD-hydrogel remained the most effective (Fig. 6d and f). This trend was further corroborated by flow cytometry analysis, which confirmed that FA@SD-hydrogel most efficiently suppressed the M1 marker CD86 and promoted the M2 marker CD163 (Fig. 6h–j).

*In vivo* findings were consistent: following HO induction, CDH5 expression decreased, the M1 macrophage marker iNOS increased, and the M2 marker CD206 decreased. Oral FA and FA@SD-hydrogel restored CDH5, reduced iNOS, and increased CD206 expression. The HO + SD-hydrogel (empty capsule) group showed no significant therapeutic effect, confirming the pharmacological inertia of the carrier. Importantly, FA@SD-hydrogel significantly restored CDH5 and improved macrophage polarization compared to oral FA, despite using only half the total drug dose (Fig. 6g, Fig. S15), with FA@SD-hydrogel outperforming oral FA. These results suggest the following mechanism in HO pathogenesis: upon stimulation, TSCs secrete MMP-2, which degrades endothelial junctional CDH5 in diseased regions, increasing local vascular permeability. This promotes migration and polarization of inflammatory cells, creating a positive feedback loop that amplifies inflammation, accelerates establishment of a local inflammatory microenvironment, and drives HO progression. FA interrupts this process by targeting MMP-2 for degradation, and our FA@SD-hydrogel system achieves this more efficiently and safely.

## 3 Discussion

HO refers to the pathological process of ectopic bone formation in soft tissues and around joints [24]. It is often secondary to severe trauma, burns, central nervous system injuries, or orthopedic surgeries [2]. The main symptoms include localized pain, swelling, and restricted joint mobility, which severely impact patients' quality of life [25]. HO is mainly classified into acquired HO and hereditary HO. Most patients have acquired HO, while hereditary HO is relatively rare, with fibrodysplasia ossificans progressiva (FOP) being a representative example [26]. Currently, nonsteroidal anti-inflammatory drugs (NSAIDs) and radiotherapy are commonly used to prevent or reduce the severity of HO [6]. However, their therapeutic effects are limited, and they carry many potential risks [8]. For patients who do not respond to conservative treatments, surgical intervention may be considered. However, surgery itself is a risk factor for HO and may lead to postoperative recurrence [19]. This study found that the MMP family plays a critical role in the pathological process of HO, particularly MMP-2, which degrades vascular endothelial CDH5 in endothelial cells. This degradation leads to the dissociation of adherens junctions between vascular endothelial cells, increased vascular permeability, and the migration of polarized M1 macrophages to the vicinity of TSCs. This exacerbates the local inflammatory microenvironment and promotes HO. The classical theory suggests that HO is primarily mediated by signaling pathways such as bone morphogenetic proteins (BMPs) and hypoxia-inducible factors (HIFs) [27]. Recent studies have shown that the matrix metalloproteinase (MMP) family, represented by MMP-9, plays a critical role in HO. MMP-9 is believed to induce the differentiation of osteoblast precursor cells and promote endochondral ossification in HO [28]. However, many studies have shown that bone formation is not completely inhibited after suppressing MMP-9 expression. The incomplete inhibition of bone formation may be partially attributed to the activation of the periosteum, which does not rely on the endochondral ossification pathway. MMP-2 is an important homolog of MMP-9, and the two share some common substrates *in vitro*. They work synergistically in various disease phenotypes, such as chronic kidney disease, type 2 diabetes, and Alzheimer's disease [29]. MMP-2 is thought to be involved in intramembranous ossification. Therefore, MMP-2 may also play an important role in HO, but its specific mechanism of action remains unclear. Network pharmacology is a pharmacological research method based on computer and network technologies. It analyzes and mines large amounts of drug molecular structure and bioactivity data, simulates and predicts interactions between drugs and targets, as well as drugs and diseases, and constructs drug networks to study drug mechanisms of action. Using network pharmacology, we identified MMP-2 as a potential target linking FA and HO. Overexpression of MMP-2 *via* plasmid transfection induced significant osteogenic differentiation in TSCs, while knocking down MMP-2 reduced osteogenic differentiation. Forsythoside A (FA) is one of the main active components of the traditional Chinese medicine Forsythia [13,14]. It exhibits pharmacological activities such as anti-inflammatory, antioxidant, antiviral, and antibacterial effects, mediates various inflammatory factors, and regulates immune responses [15,30]. Numerous studies have confirmed that FA exerts various pharmacological effects by regulating multiple signaling pathways, including NF-κB, MAPK, and JAK/STAT [31,32]. In titanium-induced inflammation of THP-1 macrophages, FA exhibited significant anti-inflammatory effects at various concentrations [[33], [34], [35]]. FA can inhibit IKK-α and IKK-β kinases, suppressing downstream NF-κB signaling activation and the release of inflammatory cytokines [36].

This study found that FA can target and inhibit MMP-2 expression, with its inhibitory effect at a concentration of 400 μg/mL being significantly higher than at 200 μg/mL. However, higher concentrations of FA exhibited cytotoxicity *in vitro* at 24 h, 48 h, and 72 h, causing drug-induced damage to vital organs such as the heart, liver, spleen, lungs, kidneys and stomach in rats. To address the side effects of oral FA, we designed an oral hydrogel loaded with FA. This hydrogel was synthesized using MA-CMCS through an amination reaction, encapsulating FA and SD. MA-CMCS has been reported as a drug carrier in various applications, including cancer therapy, cartilage repair, and wound healing [20]. The double-network hydrogel formed by MA-CMCS and oxidized locust bean gum exhibits excellent antibacterial properties, anti-inflammatory characteristics, and cell compatibility, promoting chondrocyte proliferation. FA@SD-hydrogel improved the encapsulation efficiency of FA, significantly reduced the total dosage of FA, and markedly decreased its side effects. Moreover, FA@SD-hydrogel achieved pH- and intestinal enzyme-triggered degradation in the intestine. The inherent properties of CMCS and the embedding capability of SD in the intestine ensured the intestinal retention of FA@SD-hydrogel, ultimately enabling the long-term stable release of FA. It is worth noting that while the *in vitro* conditions (pH 7.4 and standard lysozyme concentration) provide a reliable benchmark to verify the enzyme-responsive mechanism consistent with previous established models [20], they represent a simplified simulation. The actual rodent intestinal environment exhibits dynamic pH fluctuations (pH 6.5–7.0) and variable enzyme levels [37]. Nevertheless, the subsequent *in vivo* pharmacokinetic and therapeutic evaluations robustly validate the functional intestinal delivery and efficacy of the FA@SD-hydrogel system.

In summary, this study developed an oral hydrogel system, FA@SD-hydrogel, loaded with FA and SD. This system enables sustained release of FA, which targets and inhibits MMP-2–mediated CDH5 degradation, thereby suppressing the inflammatory microenvironment in tendons and offering a novel therapeutic strategy for the clinical management of HO. Nevertheless, certain limitations should be acknowledged. The investigation of FA's therapeutic target in HO focused exclusively on MMP-2 and its downstream mechanisms, without exploring potential effects on MMP-9, which may introduce confounding factors from MMP-9 and its associated signaling pathways. Additionally, the therapeutic efficacy of FA was evaluated only in small animal models, and its effects in large animal models remain to be elucidated. These aspects warrant further investigation in future studies.

## Ethical statement and clinical specimens

All animal procedures were approved by the Ethics Committee of Shanghai Tenth People's Hospital (Approval No. SHDSYY-2024-3825-3) and conducted in strict accordance with institutional regulations to minimize animal suffering and the number of animals used. Similarly, the collection of human specimens was approved by the Ethics Committee of Shanghai Tenth People's Hospital (Approval No. SHSY-LYZX-681). Heterotopic bone tissues were harvested from the elbow joints of patients diagnosed with HO group, while normal bone and adjacent tissues from patients without HO served as controls (NC group).

## Availability of data and materials

The data underpinning the findings of this research can be made available by the corresponding author upon a reasonable request.

## CRediT authorship contribution statement

**Junchao Huang:** Data curation, Formal analysis, Investigation, Methodology, Resources, Software, Validation, Visualization, Writing – original draft, Writing – review & editing. **Jinxi An:** Data curation, Formal analysis, Methodology, Software. **Le He:** Conceptualization, Data curation, Formal analysis, Funding acquisition, Project administration, Validation, Visualization, Writing – original draft, Writing – review & editing. **Huajun Wang:** Validation, Visualization. **Jiachang Hong:** Data curation, Formal analysis. **Ziheng Bu:** Investigation, Methodology, Resources, Software, Validation, Visualization. **Xudong Zhang:** Software. **Wei Liu:** Investigation. **Tingyu Wu:** Supervision, Validation. **Seng Wang:** Validation, Visualization. **Wei Zhu:** Data curation, Formal analysis, Validation, Visualization. **Yuhui Li:** Investigation, Methodology, Resources, Software. **Jixian Wan:** Investigation, Methodology. **Min Sun:** Conceptualization, Funding acquisition, Investigation, Methodology, Project administration, Resources, Software, Supervision, Writing – original draft, Writing – review & editing. **Jianzhong Du:** Conceptualization, Funding acquisition, Project administration, Supervision, Writing – review & editing. **Peng Wu:** Conceptualization, Funding acquisition, Project administration, Supervision, Validation, Visualization, Writing – review & editing.

## Declaration of competing interest

The authors declare that they have no known competing financial interests or personal relationships that could have appeared to influence the work reported in this paper.

## Data Availability

Data will be made available on request.
