# Supplementary material for: Treatment of heterotopic ossification via inhibiting the MMP-2/CDH5 axis through oral delivery of network pharmacology-predicted Chinese medicine
Source: Mater Today Bio. 2026 Apr 9;38:103107. doi: 10.1016/j.mtbio.2026.103107 (PMC13145377; doi:10.1016/j.mtbio.2026.103107)
Supplement: Multimedia component 1 [file mmc1.pdf]

Treatment of Heterotopic Ossification via Inhibiting the MMP-2/CDH5 Axis through Oral Delivery of Network Pharmacology-Predicted Chinese Medicine

Junchao Huang[a,#], Jinxi An[a,#], Le He[b,#], Huajun Wang[c,#], Jiachang Hong[a], Ziheng Bu[a], Xudong Zhang[a], Wei Liu[a], Tingyu Wu[a], Seng Wang[d], Wei Zhu[a], Yuhui Li[d], Jixian Wan[d], Min Sun[b,*], Jianzhong Du[a,b,*], Peng Wu[a,*]

[a] Department of Sports Medicine, Shanghai East Hospital, School of Medicine, Tongji University, Shanghai 200120, P. R. China

[b] School of Materials Science and Engineering, East China University of Science and Technology, Shanghai 200237, P. R. China

[c] Department of Sports Medicine, The First Affiliated Hospital, The Guangzhou Key Laboratory of Precision Orthopedics and Regenerative Medicine, Guangdong Provincial Key Laboratory of Speed Capability, Jinan University, Guangzhou, Guangdong, 510630, P. R. China

[d] Department of Orthopedics, Shanghai Tenth People's Hospital, School of Medicine, Tongji University, Shanghai 200072, P. R. China

[#] These authors contribute equally to this work.

[*]Corresponding Authors: minsun@tongji.edu.cn (Min Sun); jzdu@tongji.edu.cn (Jianzhong Du); 1700019@tongji.edu.cn (Peng Wu)

Contents

1. Supplementary methods

2. Supplementary Figures (Figures S1 – S15)

1. Supplementary methods

1.1 Acquisition of HO- and FA-Related Genes

Initially, we identified target genes related to FA by querying the SwissTargetPrediction database. Subsequently, we obtained and downloaded genes related to HO from the Comparative Toxicogenomics Database (CTD), ensuring that the data were specifically relevant to humans. The relevant data can be accessed at http://ctdbase.org/.

1.2 PPI Network Construction and Topological Analysis

In this experiment, the online tool STRING was used to analyze the protein-protein interactions (PPI) among candidate genes, with the confidence threshold (combined score) set to > 0.4. Based on the established PPI pairs, Cytoscape was used to analyze the topological structure of the PPI network. Observations of the constructed biological networks indicated that they primarily follow the characteristics of scale-free networks. Therefore, by using degree analysis in network statistics, key nodes actively involved in protein-protein interactions, particularly hub genes, can be identified in the PPI network. Additionally, this study performed node analysis and utilized the scale-free properties of the PPI network to identify hub proteins within the network.

1.3 Enrichment Analysis

R software package WebGestaltR (V0.4.4) was used for KEGG pathway analysis and GO functional enrichment analysis of differential genes, $p < 0.05$. The biological process (BP), cell component (CC), molecular function (MF), and pathways were screened in descending order according to the enrichment degree of the target. Finally, the data was visualized.

1.4 MCODE Analysis

Metascape was used for modular network analysis. Mature MCODE algorithm was used to find a few closely connected protein groups in a large and complex target network, and the biological functions of each protein group were also labeled.

1.5 Molecular Docking

This study utilized AutoDock Vina software to analyze in detail the binding affinity and interaction patterns between FA and key target genes. The molecular structure data of FA were obtained from the PubChem compound database. Meanwhile, the three-dimensional coordinate data of the hub genes were retrieved from the PDB database. To facilitate docking analysis, all protein and molecular files were converted to PDBQT format, with all water molecules removed and polar hydrogen atoms added. The grid box was centered to cover the region of each protein, allowing the molecules to move freely within it. The grid box dimensions were set to 30 Å × 30 Å × 30 Å, centered on the active site of each target protein, allowing the molecules to move freely within it.

1.6 Cell Isolation and Culture

Tendon Stem Cell Isolation: Under sterile conditions, bilateral Achilles tendons were excised from 6-8-week-old rats (150-200 g), and the tendon sheath and surrounding tissues were removed. The remaining tissue was minced into 1 mm × 1 mm pieces and digested with 3 mg/mL type I collagenase at 37 °C for 1–2 h. Digestion was terminated by adding complete medium, and the tissue was triturated into a single-cell suspension. The suspension was collected and centrifuged at 500 g for 5 minutes. The supernatant was discarded, and the pellet was resuspended in complete medium (low-glucose DMEM + 10% FBS). Cells were seeded into culture dishes, with the medium changed after 3 days and subsequently every 3 days until 80% confluence was reached. Cells were then digested and expanded for experimental use.

Endothelial Cell Isolation: Rats (6-8 weeks old, 150-200 g) were euthanized by cervical dislocation and soaked in 75% ethanol for 5 minutes. Under sterile conditions, the neck and thoracoabdominal cavities were opened, and the aorta was identified under a dissecting microscope. The aorta was placed in pre-cooled PBS, and fat and fibrous tissues were removed. The vessel was washed twice with PBS, longitudinally opened, and cut into 1 mm^3 pieces under a dissecting microscope. The tissue was digested with 0.1% type I collagenase at 37 °C for 30 minutes, and digestion was terminated by adding serum. The tissue was triturated, filtered through a 150-mesh

sieve, and the cell suspension was collected and centrifuged at 1500 rpm for 5 minutes. Cells were resuspended in endothelial-specific medium, counted, and adjusted to a concentration of 1 × 10^5/mL. Cells were seeded into 6 cm culture dishes pre-coated with type I collagen. The first 3 days were static culture, followed by medium changes every 2.5 days. After approximately 2 weeks, cells were expanded for experimental use.

Bone Marrow-Derived Macrophages Isolation: Rats (6-8 weeks old, 150-200 g) were euthanized by cervical dislocation and soaked in 75% ethanol for 5 minutes. Under sterile conditions, the femur and tibia were excised, and muscle tissues on the bone surface were removed. The bones were washed twice with PBS, and both ends were carefully cut off. Using a 5 mL syringe filled with 1640 medium, the bone marrow cavity was slowly flushed until it appeared white. The flushed bone marrow cells were collected and triturated into a single-cell suspension. The suspension was centrifuged at 1200 rpm for 5 minutes, and the supernatant was discarded. Approximately 5 mL of complete medium supplemented with 20 ng/mL M-CSF was added, and the cells were gently resuspended into a single-cell suspension. The cell density was adjusted to 1 × 106/mL, and cells were seeded into 10 cm culture dishes. After 48 h, the medium was completely replaced, and cells were cultured for an additional 3 days, for a total induction period of 5 days. On day 5, the culture dishes were washed with PBS, and the remaining adherent cells were macrophages, which were expanded for experimental use.

Rat Intestinal Epithelial cells (IEC-6) acquisition：Rat Intestinal epithelial cells were obtained from sunncell Co. SNL-318 (Wuhan, China). All cells were cultured in an incubator at 37 °C with 5% CO2. Tendon stem cells were induced to undergo osteogenic differentiation in osteogenic induction medium. The osteogenic induction medium used in this study included 10 nM dexamethasone, 50 μg/mL ascorbic acid, 10 mM β-glycerophosphate disodium, 10% fetal bovine serum, and high-glucose Dulbecco's Modified Eagle Medium (DMEM, Gibco). Inflammatory stimulation was simulated using IL-1β, with an in vitro concentration of 10 ng/mL.

Preparation of Conditioned Media (DSM and CM): To mimic the full pathological

cascade of HO in vitro, directly stimulated media (DSM) and conditioned media (CM) were prepared.

For DSM, standard culture medium was directly supplemented with inflammatory cytokines (10 ng/mL IL-1β or LPS) before applying to the target cells.

For CM, a sequential collection method was employed: primarily, TSCs were stimulated with 10 ng/mL IL-1β for 24 h. The cell-free supernatant was collected, centrifuged at 1500 rpm for 5 min to remove cell debris, and used to culture endothelial cells (ECs) for another 24 h. Subsequently, the supernatant from these ECs was harvested, centrifuged, and defined as the final CM to stimulate bone marrow-derived macrophages (BMDMs) for downstream functional assays.

1.7 Cytotoxicity Test

Cell proliferation was measured using the Cell Counting Kit-8 (CCK-8, BioSharp, BS350B) according to the manufacturer's instructions. Initially, tendon stem cells were cultured under optimal conditions until they reached an appropriate confluence level. Subsequently, these cells were co-incubated with different concentrations of FA, specifically 12.5 μg/mL, 25 μg/mL, 50 μg/mL, 100 μg/mL, 200 μg/mL, 400 μg/mL, and 800 μg/mL. After 24 h, 10 μL of CCK-8 solution was added to each well and incubated for 3 h. Absorbance readings were then precisely obtained using a spectrophotometer at a wavelength of 450 nm. Cell viability was represented as the mean ± SD of three replicates.

To evaluate intestinal safety, rat intestinal epithelial cells (IEC-6) were employed. IEC-6 were incubated with SD-hydrogel or FA@SD-hydrogel at various concentrations (125–4,000 μg/mL) for 24 h, followed by CCK-8 assay to determine cell viability. Cell viability was represented as the mean ± SD of three replicates.

For FA@SD-hydrogel, the concentration was calculated based on the mass of the hydrogel matrix alone, excluding the weight of the loaded FA.

1.8 In vitro FA Treatment Protocols (FA SSD and FA MFD)

To evaluate the therapeutic efficacy of FA and simulate the sustained-release profile of the hydrogel in vitro, two different FA administration protocols were designed:

FA SSD, defined as a Single Standard Dose: Cells were treated with a single bolus of

FA at an effective concentration of 200 μg/mL at the onset of the culture period, with no further drug supplementation.

FA MFD, defined as Multiple Fractional Doses: To mimic the continuous release of FA@SD-hydrogel, the total equivalent dose of FA was divided into equal fractions and administered sequentially at specific intervals. Specifically, 50 μg/mL of FA was added every 12 hours for a total of four applications over a 48-hour period.

1.9 Alkaline Phosphatase (ALP) Staining and Alizarin Red Staining (ARS)

Cells were subjected to alkaline phosphatase staining after 14 days of osteogenic induction. A working solution was prepared by combining 40 μL of Reagent A with 1 mL of reaction buffer, mixing thoroughly, and then adding 40 μL of Reagent B to complete the reaction mixture (Beyotime Biotechnology, PR1100). Each well was washed twice with 1 mL of PBS, with each wash lasting 1 minute. After washing, 500 μL of fixative was added to each well, and the cells were fixed at room temperature for 30 minutes. The prepared reaction mixture was distributed to each well, allowing the cells to develop color at room temperature for 30 minutes. Each well was washed three times with 1 mL of PBS, and the cells were observed and photographed under a microscope.

Cells were subjected to Alizarin Red staining after 21 days of osteogenic induction. Each well was washed with 1 mL of PBS for 1 minute. Cells were then fixed with 1 mL of 70% ethanol or 10% neutral buffered formalin at 37 °C for 30 minutes. After removing the fixative, each well was washed with 1 mL of washing solution. Subsequently, 3 mL of 1% Alizarin Red solution (Solarbio, G1452) was added to each well at room temperature and incubated at room temperature for 15–20 minutes to stain the cells. After staining, the cells were washed twice with washing solution and observed under a microscope.

1.10 Transfection

In this experiment, we used Lipofectamine 2000 reagent (Thermo, 11668-019, CA, USA) to transfect tendon stem cells to study the function of MMP-2. First, we prepared MMP-2 overexpression plasmids and siMMP-2 small interfering RNA (siRNA) and mixed them with the transfection reagent according to the

manufacturer's instructions to form transfection complexes. Subsequently, the complexes were added to tendon stem cells at final concentrations of 2 μg/mL for the overexpression vector and 50 pmol/mL for siRNA to achieve effective transfection. After transfection, cells were continuously cultured for 48 h in medium containing 10% FBS to maintain growth and transfection efficiency. Transfection efficiency was assessed using fluorescence microscopy and molecular biology methods. After 48 h, cells were collected for further experiments.

1.11 Real-time PCR Analysis

Total RNA was extracted and purified from treated cells using FreeZol Reagent (Vazyme R711-01-02). RNA concentration was measured using a NanoPhotometer (Implen, Germany). RNA was then reverse transcribed into cDNA using the 1st Strand cDNA Synthesis Kit (Crondabio KCD-M1003). Real-time PCR analysis was performed using SYBR qPCR Master Mix (Universal) (Crondabio KCD-M1004) according to the manufacturer's instructions. Glyceraldehyde-3-phosphate dehydrogenase (GAPDH) was used as an internal control.

The primers involved in this study are shown in the following table.

Genes	Sequence 5' -3'
SOX9-RT-F	CGGAACAGACTCACATCTCTCC
SOX9-RT-R	GCTTGCACGTCGGGTTTTGG
OCN -F	CTGACCTCACAGATCCCAAGC
OCN -R	TGGTCTGATAGCTCGTCACAAG
RUNX2 -F	GACTGTGGTTACCGTCATGGC
RUNX2 -R	ACTTGGTTTTTCATAACAGCGGA
GAPDH -F	GGTGAAGGTCGGTGTGAACG
GAPDH -R	CTCGCTCCTGGAAGATGGTG

1.12.FA@SD-hydrogel

Photocrosslinker MA-CMCS was prepared according to previous reports. Briefly,

biological reagent carboxymethyl chitosan (CMCS, 8.0 g) was added to 200 mL of double distilled water (DDW) and heated to 80 °C to form a clear solution, 10.0 mL of methacrylic anhydride was then added drop by drop, and the pH value was maintained at 8.0 during reaction. After 24 h of magnetic stirring under ice bath, the solution was concentrated and purified by dialysis against DDW. The white MA-CMCS powder was obtained by freeze-dried. Lithium phenyl-2,4,6-trimethylbenzoylphosphinate (photoinitiator LAP) as biocompatible free radical photoinitiator was used to prepare MA-CMCS hydrogel. After dissolving MA-CMCS at 40 mg/mL, photoinitiator LAP was added at a final concentration of 0.5% (w/v) and stirred under vacuum to a mixed well solution. Sodium deoxycholate as a small molecule penetration enhancer was uniformly mixed with the chitosan precursor solution, and was encapsulated in the hydrogel along with the formation of gel. Ultraviolet electric light was used to induce the solution to form hydrogel by irradiation at a lamp distance of 2 cm for 7 min, and then washed with DDW three times. In addition, the Forsythoside A-loaded hydrogel was prepared by adding Forsythoside A solution into preformed hydrogel solution, and the photoinduced gelation process was similar to above experiment. The synthesis of MA-CMCS and the optimized concentration of SD incorporated into the hydrogel were adopted from previously established and biologically validated protocols [DOI: 10.1007/s10118-022-2726-0].

1.13 Immunofluorescence

Frozen tissue sections were used for immunofluorescence staining. The sections were dehydrated in sucrose solutions of different concentrations and sectioned using a cryostat at a thickness of 7 μm. Subsequently, the sections were immersed in PBS three times for 5 minutes each, blocked with 10% goat serum at room temperature for 2 h, and incubated with various primary antibodies at 4 °C overnight. The sections were then washed three times with PBST solution and incubated with the corresponding secondary antibodies at room temperature in the dark for 1 h. After staining the nuclei with DAPI, the sections were observed and photographed under a fluorescence microscope (Olympus, Japan).

The primary antibodies used in this experiment included rabbit anti-MMP-2 antibody

(Proteintech 10373-2-AP, diluted 1:500) and rabbit anti-CDH5 antibody (Abcam ab318152, diluted 1:500). The corresponding secondary antibody was FITC-labeled goat anti-rabbit (Servicebio, GB22303).

1.14 Electron Microscopy

The morphology of the hydrogel was analyzed using scanning electron microscopy (SEM; Zeiss microscope) to observe the network structure of carboxymethyl chitosan hydrogel. The samples were washed three times with PBS and then frozen in a refrigerator. The samples were freeze-dried and sputter-coated with a thin layer of gold before observation.

1.15 Rheological Testing

The rheological properties of chitosan hydrogel were studied using a HAKKE rheometer. The viscoelasticity of the hydrogel was measured by performing strain sweep tests in oscillatory mode. The frequency was set to 1 Hz, and strain was varied from 0.1% to 10% to record the storage modulus (G') and loss modulus (G"). All tests were repeated with different samples. The gap width used was 5 mm.

1.16 Swelling Behavior of Hydrogel

Freeze-dried hydrogels were weighed (WD) and then stored in PBS buffer to allow water absorption. At different time points, excess water was removed by blotting, and the swollen hydrogel was weighed (WS). The WS/WD ratio was then calculated.

1.17 NMR and FTIR Measurements

1H-NMR spectra were recorded on a Bruker 400 MHz spectrometer. Samples were dissolved in D2O for analysis. Fourier transform infrared (FTIR) spectra were recorded using a Bruker Equinox 55 spectrometer, with a frequency range of 400 cm−1 to 4000 cm−1 and a resolution of 0.5 cm−1. Samples were thoroughly mixed with dry KBr powder and pressed into pellet form.

1.18 Sustained Release

The drug release of freeze-dried or non-freeze-dried hydrogels was analyzed. FA-loaded hydrogels under different conditions were immersed in 5.0 mL PBS buffer (with or without 0.4 mg/mL lysozyme; pH 7.4). At predetermined time intervals, 1.0 mL of solution was taken from the mixture, and the same volume of fresh PBS was

added. The withdrawn solution was directly filtered through a 0.22 μm syringe filter for subsequent HPLC analysis.

Chromatographic analysis of FA was performed on an Agilent 1200 series HPLC system (Agilent Technologies Inc., Santa Clara, USA). At room temperature, samples were separated using an Eclipse XDB-C18 column with an injection volume of 10 μL. A mobile phase consisting of acetonitrile and water in a 20/80 volume ratio was used to elute the samples at a flow rate of 1.0 mL/min. The eluate was monitored using a UV detector at 265 nm.

1.19 Ethical Statement

Animal experiments of this study was approved by the Ethics Committee of Shanghai Tenth People's Hospital. Animal experiments were conducted in strict accordance with relevant regulations. Ethical approval number: SHDSYY-2024-3825-3. The number of animals and their suffering were minimized as much as possible.

1.20 Human Tissues and Clinical Characterization

The study was approved by the Ethics Committee of Shanghai Tenth People's Hospital (Approval No. SHSY-LYZX-681). Heterotopic bone tissues from elbow joints were collected from patients diagnosed with heterotopic ossification (HO group). Normal bone and adjacent tissues were obtained from patients without heterotopic ossification as controls (NC group).

1.21 RNA Extraction and Library Construction

Total RNA was extracted using the TRIzol reagent method following the manufacturer's standard protocol. Quality control of the extracted RNA included quantification and purity assessment using a NanoDrop 2000 microspectrophotometer (Thermo Scientific, USA), and integrity evaluation using an Agilent 2100 Bioanalyzer (Agilent Technologies, Santa Clara, CA, USA). Library construction was performed with the VAHTS Universal V6 RNA-seq Library Prep kit, strictly adhering to the instructions provided. Transcriptome sequencing and subsequent bioinformatics analysis were completed by Suzhou Genewiz Biotechnology Co., Ltd. (Suzhou, China).

1.22 Transcriptome Sequencing and Differential Expression Analysis

Prepared libraries were sequenced on the Illumina NovaSeq 6000 platform, generating 150 bp paired-end reads. Each sample yielded approximately 30 million raw reads on average. The raw sequencing data in fastq format was initially processed through the fastp software for quality control filtering; low-quality reads were removed to obtain clean reads for downstream analysis. HISAT2 software was employed to align clean reads to the reference genome, followed by calculation of gene expression levels (FPKM values) and enumeration of aligned read counts per gene using HTSeq-count. Counts data from all samples were processed with R software (v 3.2.0) for principal component analysis (PCA) to evaluate the consistency of biological replicates within groups.

Screening of differentially expressed genes (DEGs) was conducted using DESeq2. Genes with a false discovery rate (q-value) less than 0.05 and fold change greater than 2 or less than 0.5 were defined as differentially expressed. Hierarchical clustering analysis of DEGs was performed using R software (v 3.2.0) to visualize expression pattern characteristics across different groups and samples. Subsequently, hypergeometric distribution testing was applied to DEGs for Gene Ontology (GO) functional annotation and KEGG pathway enrichment analysis to identify significantly enriched biological functional terms, which were illustrated via bar plots generated with R software (v 3.2.0).

1.23 Rat

The experimental group consisted of 60 male SD rats (6 weeks old, weighing 175 ± 5 g), which were purchased from Jiangsu Huachuang Xinnuo Pharmaceutical Technology Co., Ltd. First, the rats were anesthetized via inhalation of isoflurane (Orbiepharm, R510-22) for general anesthesia. After sterilizing the Achilles tendon area with alcohol, sterile drapes were applied, and a 1 cm incision was made along the Achilles tendon using a blade. Subcutaneous tissues were separated layer by layer to expose the tendon, followed by blunt dissection to avoid arterial injury.

Except for the sham surgery group (NC group), the Achilles tendons of rats in other groups were clamped 8 times using non-toothed surgical hemostatic forceps, with each clamp maintained for 10 seconds to induce a standardized crush injury. The

tendons were then left unsutured and transected in the middle. Amoxicillin (5 mg) was administered. Finally, the incision was sutured layer by layer with 4-0 silk sutures, ensuring that the Achilles tendon was not sutured. In the sham surgery group, the tendon was only exposed and then sutured.

FA@SD-hydrogel group: FA@SD-hydrogel (containing 4 mg of FA equivalent per dose) was administered by gavage once every 48 h for a total of seven doses (cumulative FA dose: 28 mg).

SD-hydrogel group: An equivalent mass of empty SD-hydrogel (FA-free) was administered by gavage once every 48 h for a total of seven doses.

Oral-FA: triple daily gavage for 14 days, cumulative 56 mg, with 1.33 mg FA per administration.

Finally, surviving and healthy rats from each group were selected for the experiment. Drug administration began 2 weeks after modeling and lasted for 2 weeks. Rats were euthanized at 4 weeks, 8 weeks, and 12 weeks post-modeling for experimental processing.

FA was dissolved in PBS. For in vivo experiments, the normal control (NC) and HO model groups received an equal volume of PBS vehicle to eliminate solvent-related bias.

1.24 H&E Staining and SOFG Staining

Rats were raised and euthanized in batches at the 4th, 8th, and 12th weeks, and rat tendon tissues were collected. The tissues were fixed in pre-cooled 10% formaldehyde at 4 °C for 48 h. Subsequently, the tissues were decalcified in 0.5 mol/L EDTA solution (pH 7.4) for 28 days. After decalcification, the specimens were embedded in paraffin. The paraffin blocks were sectioned into 5 μm slices and stained using standardized procedures with H&E staining reagents (Servicebio, G1005-1) and SOFG staining reagents (Solarbio, G2540 G1371).

1.25 Ex Vivo Intestinal Imaging

To visualize the gastrointestinal transit and retention of the delivery vehicle, fluorescein isothiocyanate (FITC) was utilized as a fluorescent surrogate payload and encapsulated into the hydrogel matrix at an equivalent feeding ratio to FA. The

FITC-loaded hydrogel (FITC-hydrogel) was prepared following the identical procedure used for the FA@SD-hydrogel. SD rats were orally administered with either free FITC solution or the FITC-hydrogel. At designated post-administration time points (3, 6, 12, 24, and 48 h), the rats were euthanized. The entire intact intestinal tracts were carefully harvested, coiled, and subsequently visualized using an in vivo imaging system to qualitatively evaluate the overall gastrointestinal transit and macroscopic retention time of the delivery system.

1.26 Intestinal Fluorescence Detection

4 h after administration, the SD rats were euthanized, and the intestines were harvested and fixed in 4% paraformaldehyde. The samples were embedded in paraffin and sectioned into 5 μm thick slices for immunohistochemistry. The sections were stained with DAPI to label nucleic acids and anti-ZO-1 antibody (Proteintech, 21773-1-AP, diluted 1:500) to label tight junction proteins. The stained sections were analyzed using a confocal laser scanning microscope (Olympus, FV3000).

1.27 Immunohistochemical Staining

Paraffin tissue sections were used for immunohistochemical staining. Antigen retrieval was performed by incubating the sections in citrate buffer (pH 6.0) at 95 °C for 20 minutes. The primary antibodies used in this experiment included anti-MMP-2 antibody (Proteintech, 10373-2-AP, diluted 1:500) and HRP-labeled goat anti-rabbit antibody (Biosharp, BL003A).

1.28 Micro CT

After 12 weeks of feeding post-modeling, micro-CT analysis was performed. The distal tibia and Achilles tendon of the calcaneus were fixed overnight in 10% formalin. The X-ray tube was set to 50 kV and 60 μA, with an image acquisition resolution of 18 μm. Images were captured at 0.5° rotation steps over a 360° range with 50 ms exposure per step. Image reconstruction and analysis were performed using Skyscan 1275 software.

1.29 Western Blotting

Protein lysates were obtained from each group using RIPA lysis buffer. Protein concentrations in the samples were determined using the BCA method. Proteins were

separated by SDS-PAGE gel electrophoresis and then transferred onto a polyvinylidene fluoride (PVDF) membrane. The PVDF membrane was blocked in 5% BSA solution at room temperature for 2 h and then incubated with primary antibodies overnight at 4 °C on a shaker. After washing with TBST solution, the membrane was incubated with secondary antibodies at room temperature for 1 h. After washing three times with TBST solution, protein bands were visualized using ECL reagents and the Tanon 5200 chemiluminescence imaging system. Grayscale values were evaluated using Image J software.

The primary antibodies used in this experiment included anti-MMP-2 antibody (Proteintech, 10373-2-AP, diluted 1:800) and anti-β-actin antibody (Servicebio, GB15001).

1.30 Cell Migration Assay

BMDMs from each group were added to Transwell chambers and incubated at 37 °C for 24 h. The upper chamber of the Transwell was gently washed 2–3 times with PBS to remove non-migrated cells. Cells were fixed with 4% paraformaldehyde for 15 minutes, stained with 0.1% crystal violet for 20 minutes, and the non-migrated cells on the upper layer were gently wiped off with a cotton swab. The chamber was washed three times with PBS. The migrated cells were observed and photographed under a microscope.

1.31 Statistical Analysis

All data were repeated three or more times ($N \geq 3$) and recorded as mean ± standard deviation (SD). Statistical significance between two or more groups was calculated using a two-tailed Student's t-test or two-way ANOVA, while comparisons among three or more groups were performed using one-way or two-way ANOVA, followed by Dunnett's test for multiple comparisons. A P-value less than 0.05 was considered statistically significant. Statistical analysis was performed using Prism 10.0 software (GraphPad Prism).

2. Supplementary Figure (Figures S1 –S15)

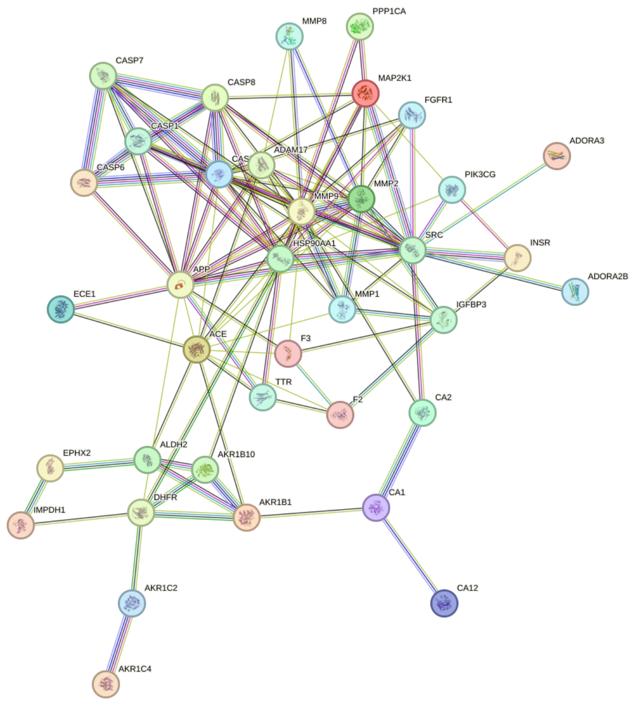

Figure S1. Protein-protein interaction network built from HO-FA overlapping genes

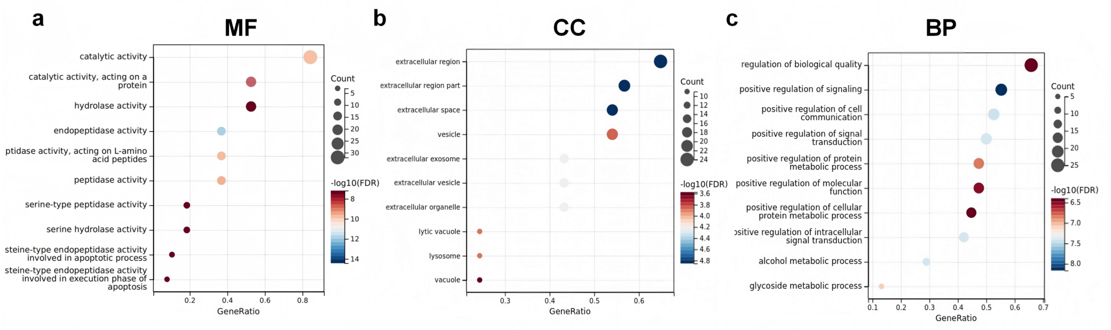

Figure S2. GO enrichment analysis of the common targets. a) Molecular Function. b) Cellular Component. c) Biological Process.

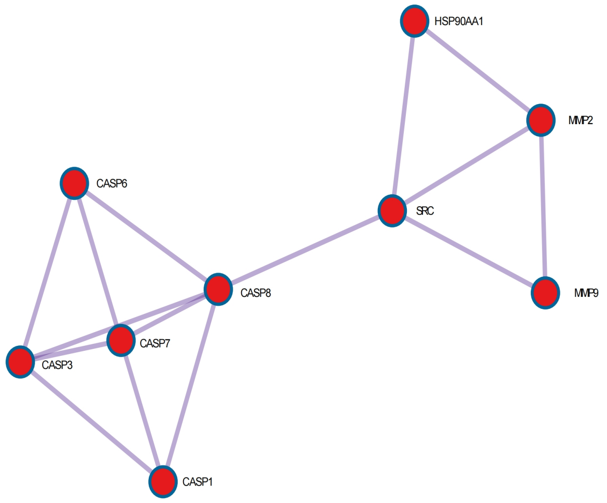

Figure S3. MCODE analysis network built from HO-FA overlapping genes.

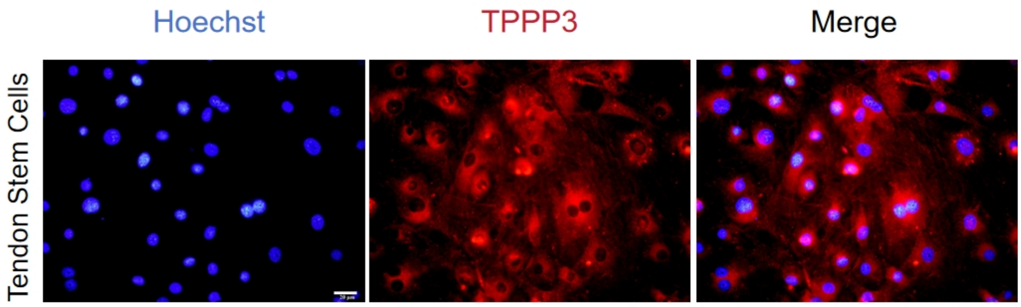

Figure S4. Representative immunofluorescence images of TPPP3 (a tendon stem cell marker) in TSCs. Nuclei were stained by Hoechst. Scale bar: 20 μm.

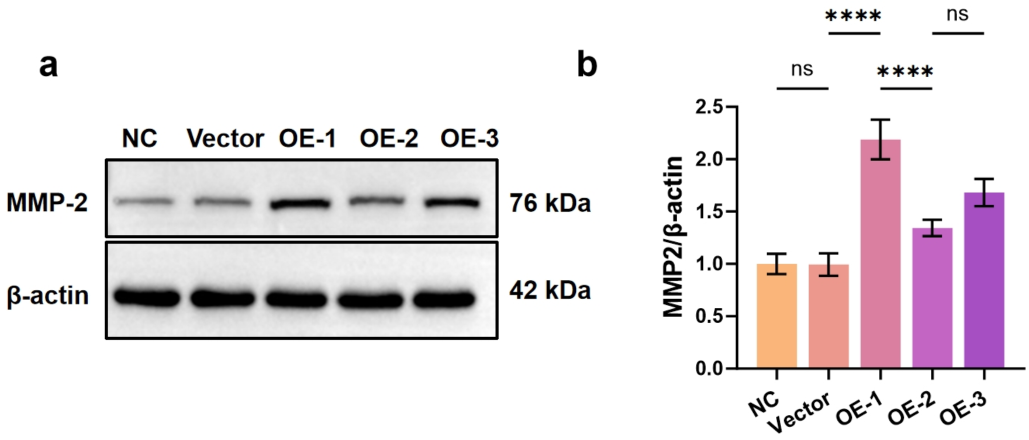

Figure S5. Validation of MMP-2 overexpression efficiency. a) Western blot analysis and b) corresponding quantification of MMP-2 protein levels in TSCs after different treatments. Data are presented as mean ± SD (N = 3)

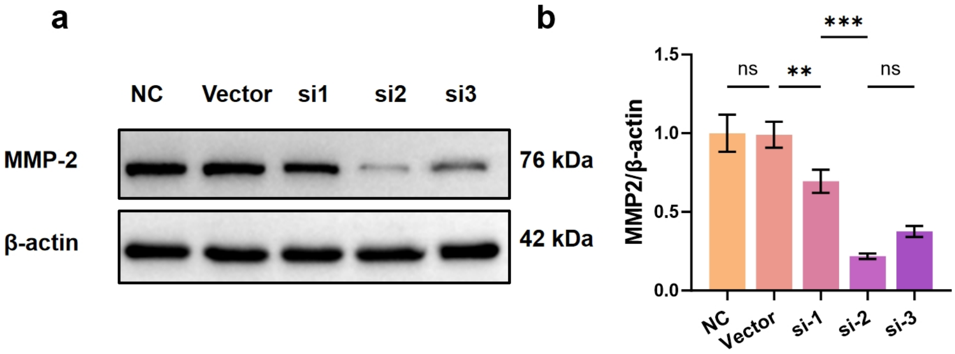

Figure S6. Validation of MMP-2 knockdown efficiency. a) Western blot analysis and b) corresponding quantification of MMP-2 protein levels in TSCs after different treatments. Data are presented as mean ± SD (N = 3)

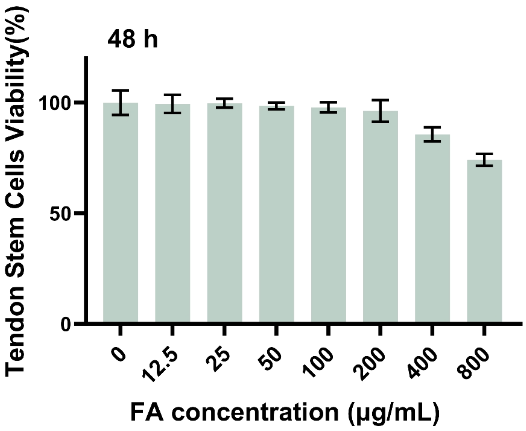

Figure S7. Viability of TSCs after 48 h of incubation with different concentrations of FA. Data are expressed as means ± SD (N = 3).

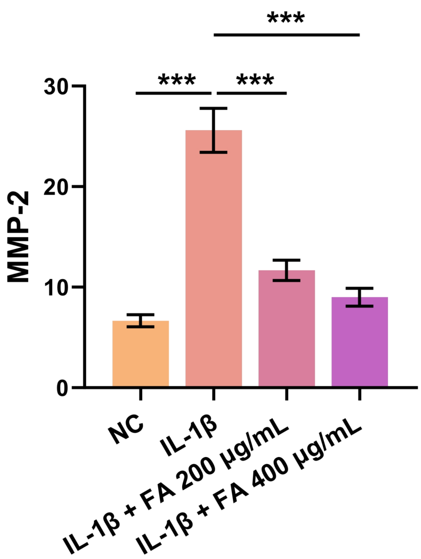

Figure S8. Quantitative analysis of MMP-2 fluorescence intensity in TSCs under the indicated treatments. Data are expressed as means ± SD (N = 3).

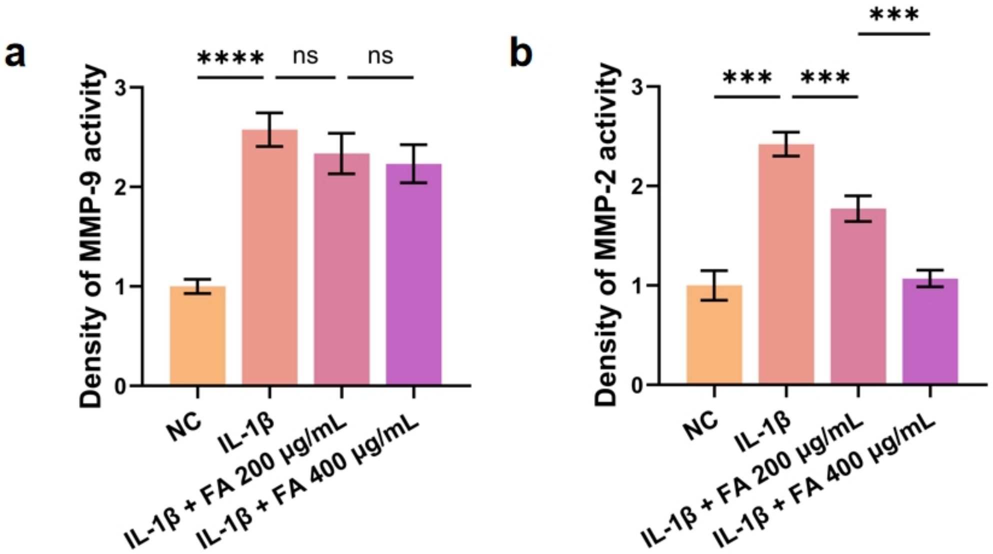

Figure S9. Quantitative analysis of MMP-9 and MMP-2 activities in TSCs under the indicated conditions. Data are expressed as means ± SD (N = 3).

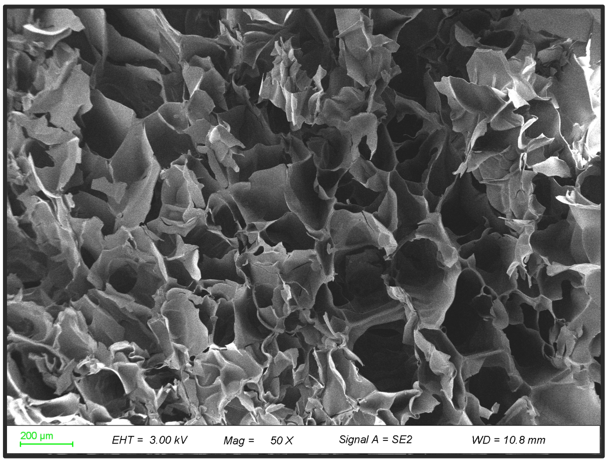

Figure S10. Scanning electron microscopy (SEM) image of FA@SD-hydrogel

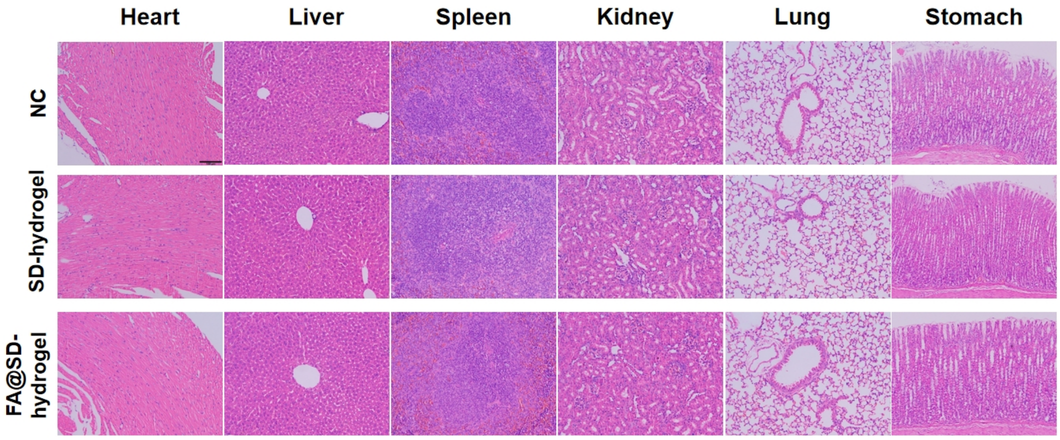

Figure S11. Representative H&E-stained images of major organs (heart, liver, spleen, lung, kidney, stomach) from rats after different treatments. Scale bar: 100 µm.

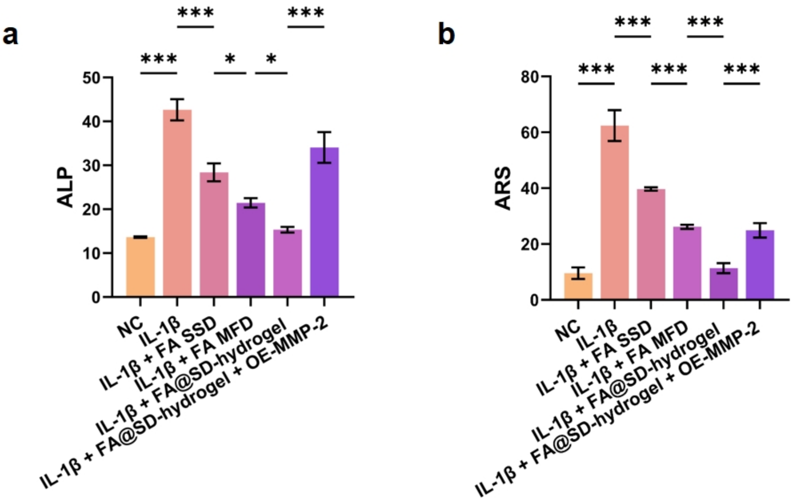

Figure S12. Quantitative analysis of a) ALP and b) ARS. Data expressed as mean ± SD (N = 3).

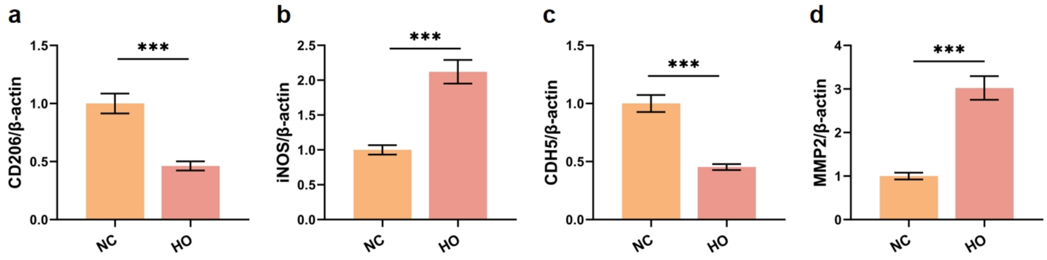

Figure S13. Quantitative analysis of a) CD206, b) iNOS, c) CDH5, and d) MMP-2 protein levels in human tissues from healthy individuals and HO patients. Data are expressed as means ± SD (N = 3).

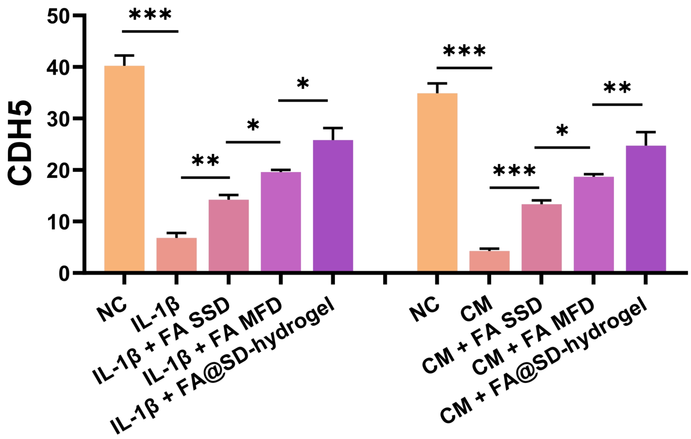

Figure S14. The corresponding quantification of CDH5 fluorescence intensity. Scale bar: 50 µm. Data are expressed as means ± SD (N = 3).

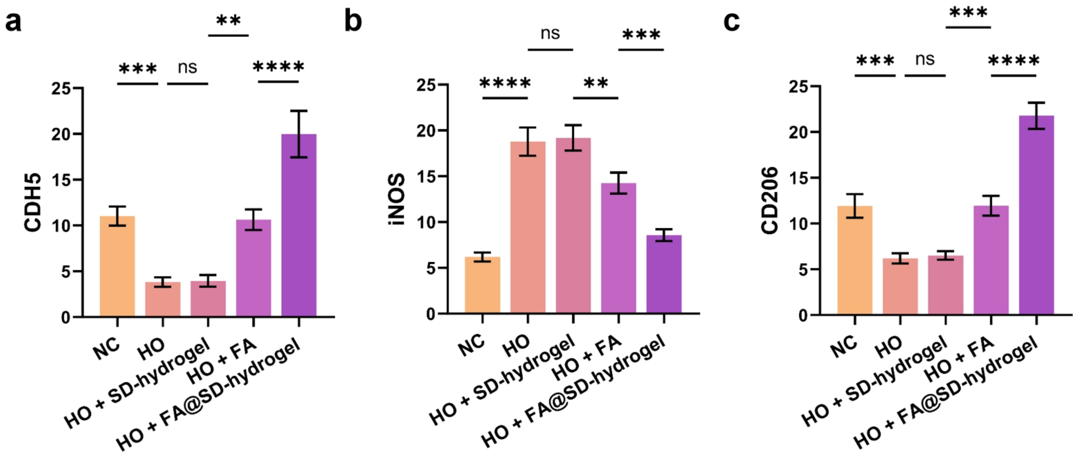

Figure S15. Quantitative immunohistochemical analysis of a) CDH5, b) iNOS and c) CD206. Data expressed as mean ± SD (N = 3).
